# Adapting a complex health intervention guided by the core functions and forms model: a methodology and case example of participatory adaptation

**DOI:** 10.3389/frhs.2026.1876032

**Published:** 2026-08-31

**Authors:** Kirsten E. Austad, Nhi Nguyen, Elizabeth Jadovich, Bridget Poznanski, Max Laguerre, Alicia Fernandez, Mari-Lynn Drainoni, Brian Jack, Suzanne Mitchell, Ramzi G. Salloum, Graham Moore

**Affiliations:** 1 Department of Family Medicine, Boston Medical Center & Boston University Chobanian & Avedisian School of Medicine, Boston, MA, United States; 2 Evans Center for Implementation & Improvement Sciences (CIIS), Department of Medicine, Boston University Chobanian & Avedisian School of Medicine, Boston, MA, United States; 3 Department of Medicine, Cambridge Health Alliance, Cambridge, MA, United States; 4 School of Social Work, Boston College, Chestnut Hill, MA, United States; 5 Department of Pediatrics, Boston Medical Center & Boston University Chobanian & Avedisian School of Medicine, Boston, MA, United States; 6 Department of Medicine, University of California San Francisco, San Francisco, CA, United States; 7 Section of Infectious Diseases, Department of Medicine, Boston Medical Center/Boston University Chobanian & Avedisian School of Medicine, Boston, MA, United States; 8 Department of Health Law Policy and Management, Boston University School of Public Health, Boston, MA, United States; 9 Department of Family Medicine and Community Health, UMass Chan Medical School, Worcester, MA, United States; 10 Department of Health Outcomes & Biomedical Informatics, University of Florida College of Medicine, Gainesville, FL, United States; 11 DECIPHer, School of Social Sciences, Cardiff University, Cardiff, United Kingdom

**Keywords:** complex intervention, core functions and forms model, hospital discharge, intervention adaptation, non-English language preference

## Abstract

Intervention adaptation—the intentional modification of a program, practice, or innovation's design or delivery—is essential for improving fit when interventions are implemented with diverse populations, yet how to adapt without compromising core mechanisms remains a persistent challenge for complex, multicomponent programs. This article describes how the Core Functions and Forms (CFF) model, used in conjunction with the ADAPT guidance, addresses this challenge by distinguishing essential intervention mechanisms (core functions) from adaptable delivery strategies (forms). To illustrate the approach, we present a case example of adapting hospital discharge interventions for patients with non-English language preference. A 13-member Adaptation Team of patients, caregivers, and hospital staff participated in four facilitated sessions to identify and prioritize core functions shared across existing discharge interventions, then propose context-appropriate forms—distinguishing specific forms tied to individual functions from cross-cutting forms spanning who delivers the intervention, when, where, and how language concordance is achieved. Rankings and impact–feasibility ratings informed deliberative prioritization, surfacing functions that balanced high impact with feasibility as well as those judged impactful but harder to implement. Applying the CFF model across a family of related interventions, rather than a single program, shows how shared mechanisms can be reused and tailored for diverse populations. This manuscript reports the adaptation-design phase; subsequent pilot testing is planned. The approach offers a transparent, replicable method for equity-focused adaptation that preserves intervention mechanisms while tailoring delivery.

## Introduction

Intervention adaptation, the intentional modification of a program, practice, or innovation's design or delivery, is a common strategy to improve an intervention's fit within specific contexts (1). When evidence-based interventions (EBIs) are implemented with diverse populations, adaptation plays a crucial role in aligning interventions with the unique needs and preferences of these groups (2–4). This alignment can enhance an intervention's implementability and effectiveness and promote health equity, making adaptation a vital component of efforts to achieve equitable health outcomes (5–8). Despite its importance, many questions remain about the best approaches to adapting interventions, particularly in high diversity contexts (9, 10).

Ensuring an EBI fits the local context is a widely accepted first step in implementation. Approaches such as ADAPT and ADAPT-ITT explicitly require assessing intervention-context fit and recommend that adaptations remain aligned with the intervention's functions (11–13). A persistent tension is tailoring interventions while ensuring that effect-producing elements are preserved. Frameworks such as Consolidated Framework for Implementation Research (CFIR) distinguish “core components” vs. “adaptable periphery” (14–18). However, in practice it is difficult to specify which elements are truly essential or to predict which adaptations will alter an intervention's mechanism of action or have unintended effects (19–21). This uncertainty is greater for complex interventions, those that feature multiple interacting components, variable implementation processes, heterogeneous outcomes, and complex interactions with context, because it is harder to measure and track which elements are responsible for the observed effects (22–24).

One approach that may address these challenges is the Core Functions and Forms (CFF) model (25–27). The CFF operationalizes ideas first advanced by Hawe and colleagues in 2004 around defining intervention integrity in terms of functions, while allowing forms to vary across context (25). CFF posits that interventions are defined by a consistent set of core functions (the essential mechanisms of an intervention to preserve), while the forms (the specific ways these functions are delivered) can and should vary based on the needs of the implementation context and population. For example, consider an evidence-based patient education intervention originally developed and tested using an English-language handout. The core function of the EBI is “to help patients understand their illness.” If one sought to adapt the EBI for a setting where many patients prefer Spanish or have limited literacy, the same function would not be achieved by an English language handout but would require a written Spanish handout, illustrated materials, or a language-concordant audio recording of the content of the written materials. Not adapting the form—e.g., providing only English text—would in fact undermine its effectiveness. By centering adaptation on functions rather than fixed components, CFF offers a principled way to resolve the fidelity-tailoring paradox which aims to preserve the mechanisms that produces benefits (the function), while permitting flexible, context-appropriate changes in delivery (the form).

A deeper understanding of these core functions and adaptable forms offers a useful conceptual model for equity-focused intervention adaptation. This manuscript aims to describe the application of the CFF model to support stakeholder-engaged intervention adaptation and analyze the benefits and drawbacks of this approach. We focus on a case study involving the adaptation of a hospital discharge EBI for patients with a U.S. safety net hospital. Specifically, we applied the CFF model within the ADAPT process to define core functions and identify context-appropriate forms for hospital discharge interventions serving NELP populations (11).

### Case example: evidence-based intervention to improve hospital discharge

The transition from hospital to home after hospital discharge is a high-risk and vulnerable period for both patients and caregivers (28). Hospital discharge processes require coordination among hospital teams, clear handoffs between inpatient and outpatient clinicians, accurate medication reconciliation, and instructions to patients and caregivers that are both understandable and actionable. Undelivered or unclear communication, ambiguous delineation of responsibilities, and unrealistic expectations placed on patients and their caregivers (29–31) are common and contribute to high readmission rates and low patient satisfaction (32). Several hospital discharge interventions have been shown to reduce hospital readmission rates and improve patient-centered outcomes, such as Project RED and the Care Transitions Intervention (33–38),. These hospital discharge EBIs exemplify complex interventions because they include multiple interacting components, generate non-linear causal pathways, vary substantially in how they are implemented across settings, and are contextually dependent (39).

An important limitation, however, is that nearly all hospital discharge EBIs have been designed for and tested only in English-fluent populations (40, 41). Individuals with non-English language preference (NELP), a growing demographic in the United States over the past 40 years (42), have been routinely excluded from hospital discharge trials, yet are at very high risk for poor outcomes due to inherent communication barriers (43, 44). As a result, these EBIs do not address language-related barriers that arise in routine practice, such as translating provider-written discharge instructions, overcoming language barriers in verbal communication, or consider other unique needs of those with NELP (45, 46). Without deliberate adaptation, deploying these EBIs in real-world, linguistically diverse settings risks reducing their effectiveness and widening health inequities (47–49). Because discharge EBIs are complex, a function-based approach is particularly useful for ensuring essential mechanisms remain intact when adapting for linguistically diverse populations.

## Methods

### Overview of approach

**Figure 1:**
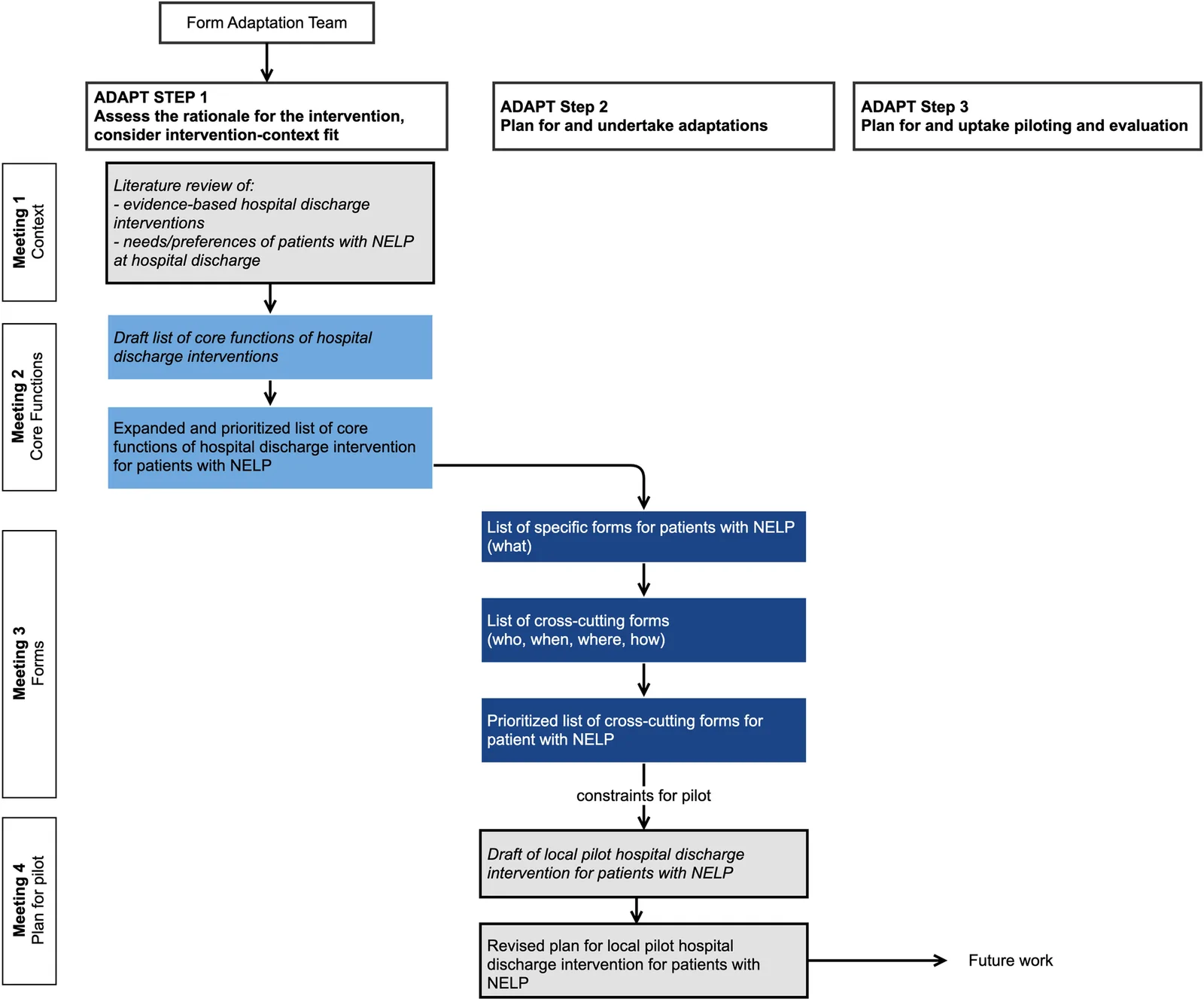
Step-by-step depiction of how the ADAPT guidance and the core functions and forms (CFF) model were integrated to adapt a hospital discharge intervention for patients with non-English language preference (NELP). Steps of the ADAPT guidance are shown left to right; content of each Adaptation Team meeting is shown top to bottom. Light blue boxes denote steps that focused on core functions and dark blue boxes those that focused on intervention forms. Tasks shown in italics were led by the study team; those in regular type were led by the Adaptation Team.

The overall goal of this study was to adapt a hospital discharge intervention for patients with NELP for future pilot testing. Our assessment of intervention-context fit (discussed above) found that no single existing hospital discharge intervention would fit the needs of populations with NELP. However, many hospital discharge EBIs share common components, such as patient education, medication reconciliation, care coordination, and post-discharge outreach (38, 39). Rather than adapting a single EBI, we therefore framed adaptation around core functions (what an intervention must achieve) rather than forms (specific program components), aiming to preserve the mechanisms that produce benefit while tailoring delivery to the needs of patients with NELP. The author team selected the ADAPT guidance to guide the process, with tasks divided between the author group (italicized steps in Figure 1) and the Adaptation Team (non-italicized steps) (11).

**Figure 2:**
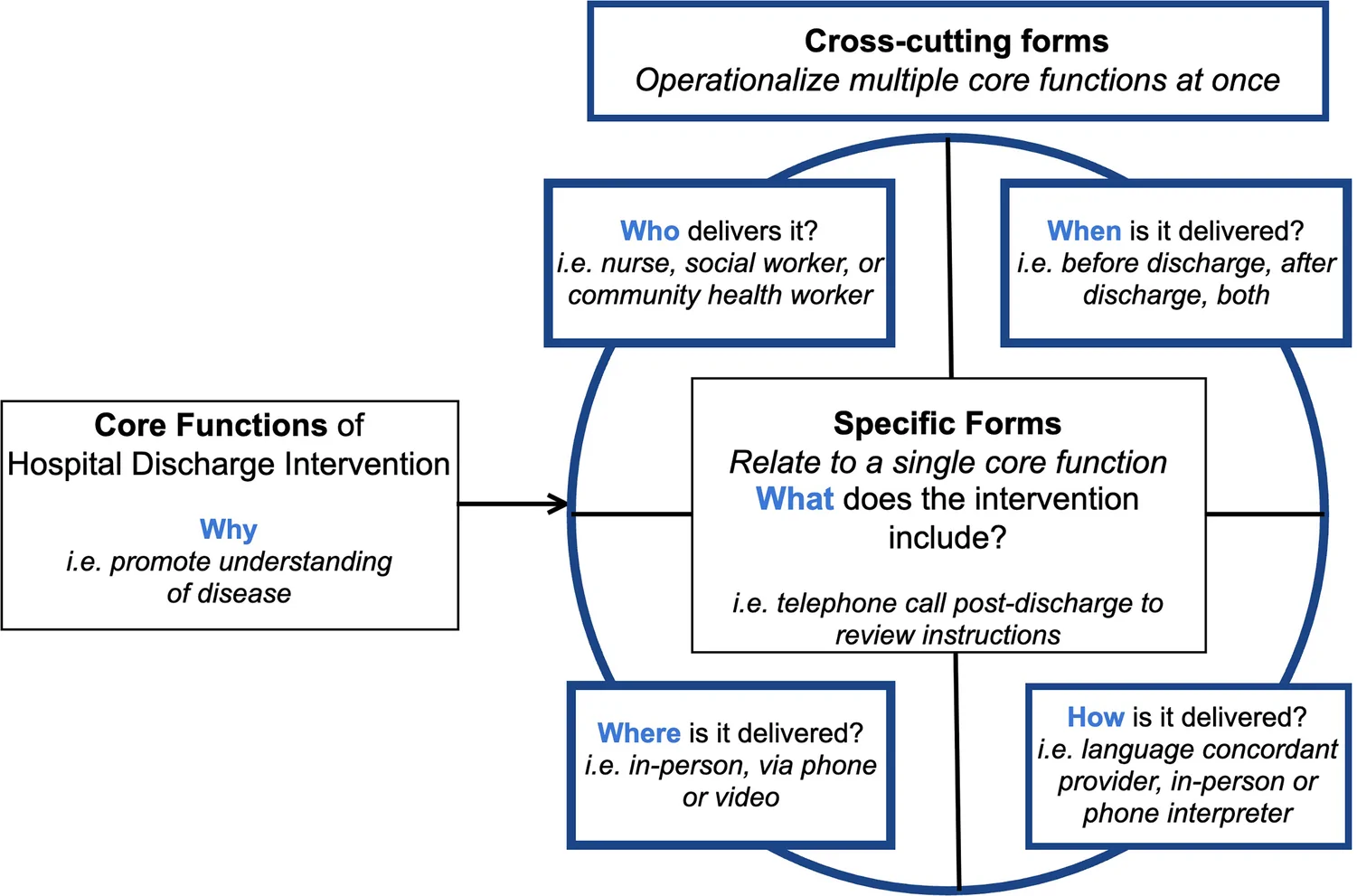
How the core functions and forms (CFF) model was applied to adapt a hospital discharge intervention for patients with non-English language preference (NELP).

The research team first established an initial list of candidate core functions through evidence synthesis. Drawing on a recent systematic review (39), we identified hospital discharge interventions conducted in the United States (excluding those limited to pediatric, psychiatric, or surgical admissions) (33, 50–82). The lead author reviewed the primary publication for each included intervention to abstract its core functions.

The Adaptation Team was given three tasks: (1) review the draft list of core functions from literature review, add core functions important for patients with NELP, and prioritize them; (2) propose context-specific adaptable forms (the activities, materials, and delivery strategies that could reliably achieve each core function in a multilingual, multicultural care context); and (3) provide iterative feedback on proposed functions and forms to inform pilot implementation. Forms were of two types: specific forms correspond to a single core function—the concrete activity, material, or strategy used to achieve it—while cross-cutting forms operationalize multiple core functions at once: who delivers the intervention, and where, when, and how it is delivered (notably, how language barriers are addressed Figure 2).

### Study setting

Study activities occurred at Boston Medical Center (BMC), the largest safety net hospital network in New England. BMC serves a diverse population, with the majority of patients identifying as Black or Latinx. Approximately one-third of all patients have NELP. This high linguistic diversity context includes a patient population that is about 54% Spanish speaking, 18% Haitian Creole speaking, 9% Cape Verdean (Portuguese) Creole speaking, 7% Vietnamese speaking, and the remainder speaking over 250 additional languages.

### Participant recruitment

We aimed to recruit 10–14 Adaptation Team members, including patient and caregiver representatives and BMC employees. Patient and caregiver representatives were eligible if they had direct experience with hospital discharge as a patient with NELP or had assisted a patient with NELP during discharge. We prioritized recruiting individuals whose first language was not English and advertised the availability of simultaneous interpretation and translation services to enable participation by monolingual individuals with NELP. To identify these representatives, we engaged community networks and used snowball sampling to reach individuals outside BMC with relevant lived experience. Non-employee participants received $125 pre-loaded debit cards for each adaptation meeting attended and for completing voting and survey activities.

We sought to include BMC employees across provider types involved in hospital discharge, including physicians, advanced practice providers, nurses, case managers, and social workers. To recruit BMC staff, we emailed supervisors in nursing, interpreter services, social work, and inpatient provider departments, who then shared study information with potentially interested team members.

### Adaptation team meetings

We convened the Adaptation Team via videoconference four times between May and June 2025, with each 90-minute session facilitated by the PI. To encourage participation, the PI used slides alongside interactive Mentimeter polls for voting/ranking and comment boxes (83). Voting exercises served to surface opinions and stimulate discussion rather than to impose hard cutoffs, as in a formal Delphi process (84). Table 2 summarizes meeting topics. Each session began with a short recap and open discussion. Team members who missed a session were offered the opportunity to meet with the PI or review the written transcript.

Meeting 1 opened with introductions and a process-mapping exercise to describe the hospital discharge process from an NELP patient's perspective, followed by a presentation of the literature review of discharge EBIs, and a group brainstorming of challenges, strengths, and implications for populations with NELP.

In Meeting 2, we presented a revised process map and then introduced the CFF model, walking participants through an everyday example to illustrate the concepts and invite questions. To reinforce the distinction between core functions and forms, we used a consistent color convention on all slides. We then reviewed the initial list of candidate core functions derived from the literature, which team members critiqued and revised drawing on their clinical and lived experience.

In Meeting 3 the group discussed their preliminary ranking and ratings of the impact and feasibility of core functions via Mentimeter polls and discussed emerging patterns. Next, they brainstormed intervention forms for each core function. For specific forms, participants brainstormed as many ideas as time allowed. The research team derived candidate cross-cutting forms from literature on commonly adapted aspects of EBIs (85); participants were asked to begin considering the feasibility and impact of each using Mentimeter polls, which laid out the groundwork for the formal voting and ranking process described below.

Meeting 4 presented voting results, facilitated discussion, and solicited feedback on the proposed pilot model based on information collected in earlier meetings within the constraints of alignment with the funded grant.

### Data collection

Demographic information for each Adaptation Team member was collected using a REDCap survey. All four meetings were audio-recorded and transcribed using Zoom's AI transcription feature. Transcripts were then reviewed by a research assistant (RA) for accuracy and identifying information was redacted to maintain participant confidentiality.

Rating by all Adaptation Team members was conducted using REDCap surveys. Members ranked a list of 15 core functions of a hospital discharge intervention for patients with NELP on a scale from 1 to 15, where 1 indicated the core function with the highest importance and 15 the lowest. Additionally, for each core function, members assigned two scores from 1 to 10: one representing the perceived impact of the core function and the other representing the feasibility of incorporating it into a hospital discharge intervention. Lower scores indicated lower impact or feasibility, respectively.

The Adaptation Team also rated the impact and feasibility of cross-cutting forms, Participants were asked to assign scores from 1 to 10 to indicate the perceived feasibility and impact across the following questions: where and when the intervention should be delivered, who should deliver the intervention, and how to overcome the language barrier.

### Data analysis

Two analysts independently reviewed the transcripts from each meeting. We used conventional content analysis with descriptive coding to extract and enumerate discrete ideas or suggestions from participants. Prior to the next meeting, these ideas were compiled into a consolidated list, which served as the basis for subsequent discussion and rating.

The research team then conducted summative analyses of the Adaptation Team's input on core functions. From the core function ranking exercise we calculated an average ranking to summarize the group's relative prioritization. Next, we computed mean impact and mean feasibility scores from the voting exercise. We produced a bubble plot to display these results with mean feasibility on the *x*-axis, mean impact on the *y*-axis, and one bubble per core function; bubble size represented standard deviation, so larger bubbles indicated greater variance in opinions. This visualization highlighted functions that combined high impact with high feasibility, as well as those judged high impact but less feasible to implement. We applied the same analytic approach to each of the crosscutting forms, generating analogous bubble plots to summarize perceived impact–feasibility tradeoffs for those elements. Lastly, we abstracted from transcripts all ideas for specific forms generated by the Adaptation Team and organized them by corresponding core function.

### Ethics

This study was approved by the Boston University Medical Center Institutional Review Board (H-44249), including a waiver of documentation of written consent. All participants provided verbal informed consent at the start of each Adaptation Team meeting.

## Results

### Adaptation team and meeting structure

In total, 13 individuals participated in the Adaptation Team. Participant demographics are provided in Table 1. All participants were fluent in English, 69% spoke at least one additional language and 54% spoke a language other than English at home. Most team members (77%), including non-BMC employees, were employed in health-care related fields. The team was made up of primarily females (92%) who were young adults 35 years or under (69%) and had previously or currently acted as caregiver for a loved one (85%).

**Table 1:** Demographics of adaptation team members.

Demographic	*N* (%)
Gender	
Male	1 (8)
Female	12 (92)
Age	
18–24 years	3 (23)
25–35 years	6 (46)
36–45 years	3 (23)
46–55 years	0 (0)
55 years or older	1 (8)
Profession	
Health care worker: physician, advanced practice provider, or resident	4 (31)
Health care worker: Interpreter	2 (15)
Health care worker: Social worker	3 (23)
Health care worker: Other	1 (8)
Other	3 (23)
Languages of fluency	
English	13 (100)
Spanish	5 (38)
Haitian Creole	3 (23)
Vietnamese	1 (8)
French	1 (8)
Language spoken most often at home	
English	6 (46)
Spanish	3 (23)
Haitian Creole	2 (15)
Vietnamese	2 (15)
Ever been hospitalized	
Yes	8 (62)
No	5 (38)
Ever acted as a caregiver	
Yes	11 (85)
No	2 (15)
Boston Medical Center employee	
Yes	7 (54)
No	6 (46)

Initially we planned to conduct voting during Meetings 2 and 3 using Mentimeter. However, based on Adaptation Team feedback requesting more time to deliberate—both on the distinction between core functions and forms and on ranking choices—we moved to asynchronous polling after Meeting 3, distributing polls via email (Table 2). Attendance was relatively consistent throughout the course of the adaptation process with the number of Adaptation Team members in attendance ranging from 8 to 11.

**Table 2:** Overview of adaptation team meetings and activities.

Date	Description	*N*	Goals and Agenda
05/06/2025	Meeting 1 Virtual	10	Introduction of meeting participants Build a shared understanding of current discharge process (part I) Literature review on evidence-based hospital discharge interventions and patients with NELP at hospital discharge Brainstorm needs and strengths of patients with NELP relevant to hospital discharge
05/13/2025	Meeting 2 Virtual	11	Review summary of last session and interim input Build a shared understanding of current discharge process (part II) Explanation of Core Functions and Form model (using every day example) Discuss list of core functions and brainstorm additional ones needed for populations with NELP
05/20/2025	Meeting 3 Virtual	8	Review summary of last session and interim input Brainstorm forms of a hospital discharge intervention for patients with NELP including: (1) Forms that correspond to a single core function (the “what”) (2) Cross-cutting forms that specify the means of delivery of other forms (the “who, when, where, and how”)
	*Voting Asynchronous	9	Rank importance of core functions Rate feasibility and impact of each core function Rate feasibility and impact of the crosscutting forms
06/02/2025	Meeting 4 Virtual	10	Review results of group voting Open discussion of proposed design of pilot intervention at Boston Medical Center

CFF, Core functions and forms model; NELP, Non-English language preference.

* Initially planned on voting during Meeting 2 and 3 however based on feedback from Adaptation Team members this was made asynchronous after Meeting 3 to allow further time for deliberation.

### Ranking of core functions

In the ranking exercise, participants ranked “understanding and addressing priorities of the patients” as the most important core function (mean rank = 13.7, out of 15) followed by “assess and address linguistic and cultural needs” (mean rank = 11.8) and “involve caregiver” (mean rank = 10.9). Lower ranked core functions included “promoting self-care” (mean rank = 5.1), “ensure transmission of information to primary care provider” (mean rank = 4.3), and “promote inpatient continuity” (mean rank = 2.1).

### Rating core functions

**Figure 3:**
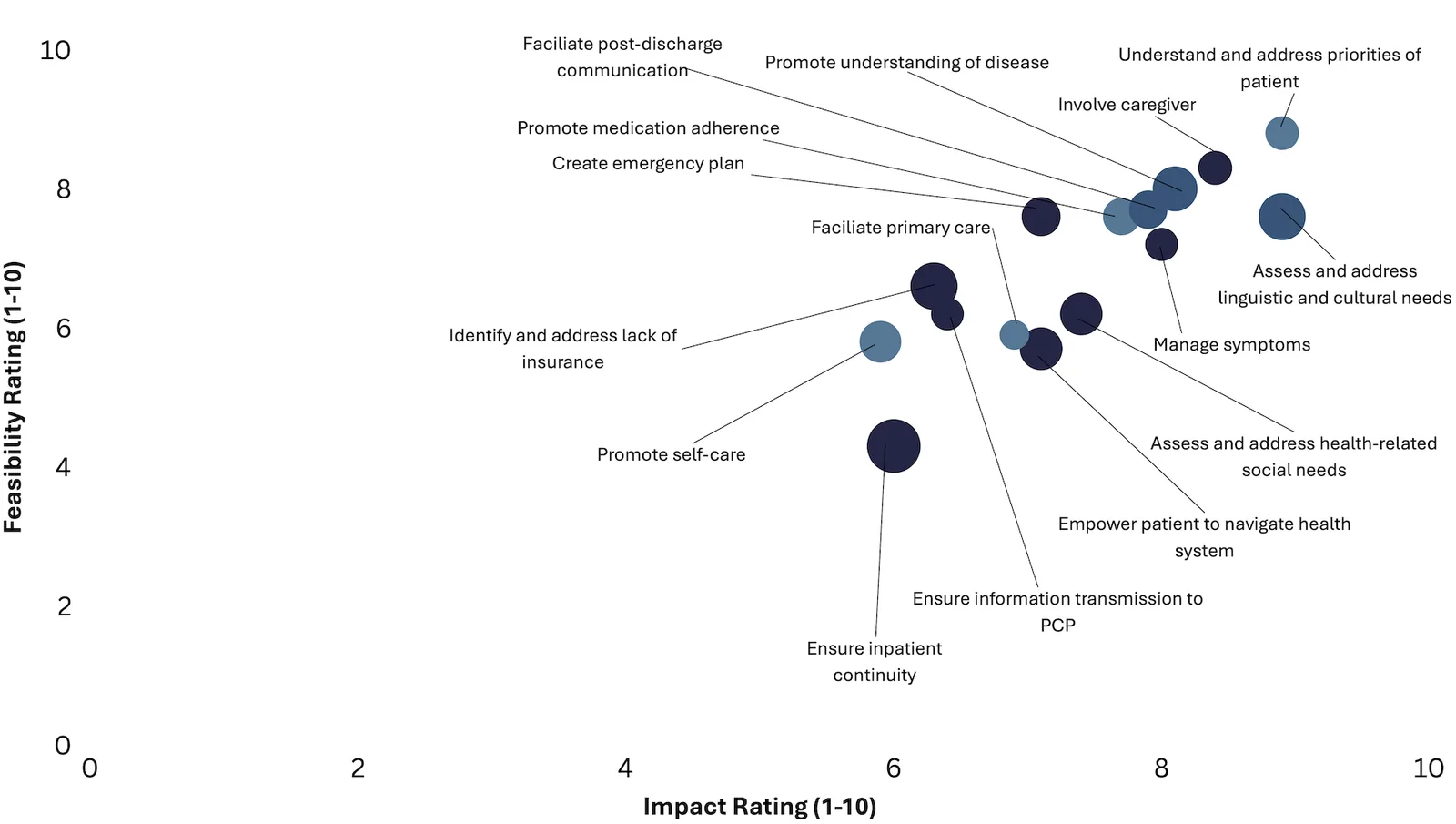
Quadrant plot of feasibility and impact ratings for core functions of hospital discharge interventions for patients with non-English language preference (NELP). Each bubble represents one core function, positioned by mean feasibility (*x*-axis) and mean impact (*y*-axis). Bubble size reflects the standard deviation, with larger bubbles indicating greater variance in ratings.

When Adaptation Team members rated the impact of each core function, “understand and address patient priorities” and “involve caregivers” received the highest impact ratings (mean impact score = 8.9 for both, out of 10) and were also rated as highly feasible (mean feasibility scores = 8.8 and 8.3, respectively). The next most impactful core function was “assess and address linguistic and cultural needs” (mean impact score = 8.4), but slightly less feasible (mean feasibility score = 7.6). Core functions rated as having low feasibility of achieving included “ensuring inpatient continuity” (mean feasibility score = 4.3), “empower patient to navigate the health system” (mean feasibility score = 5.7), and “promote self-care” (mean feasibility score = 5.8). These results are summarized in the bubble plot in Figure 3 (ranking in Supplementary Appendix).

### Rating forms

**Figure 4:**
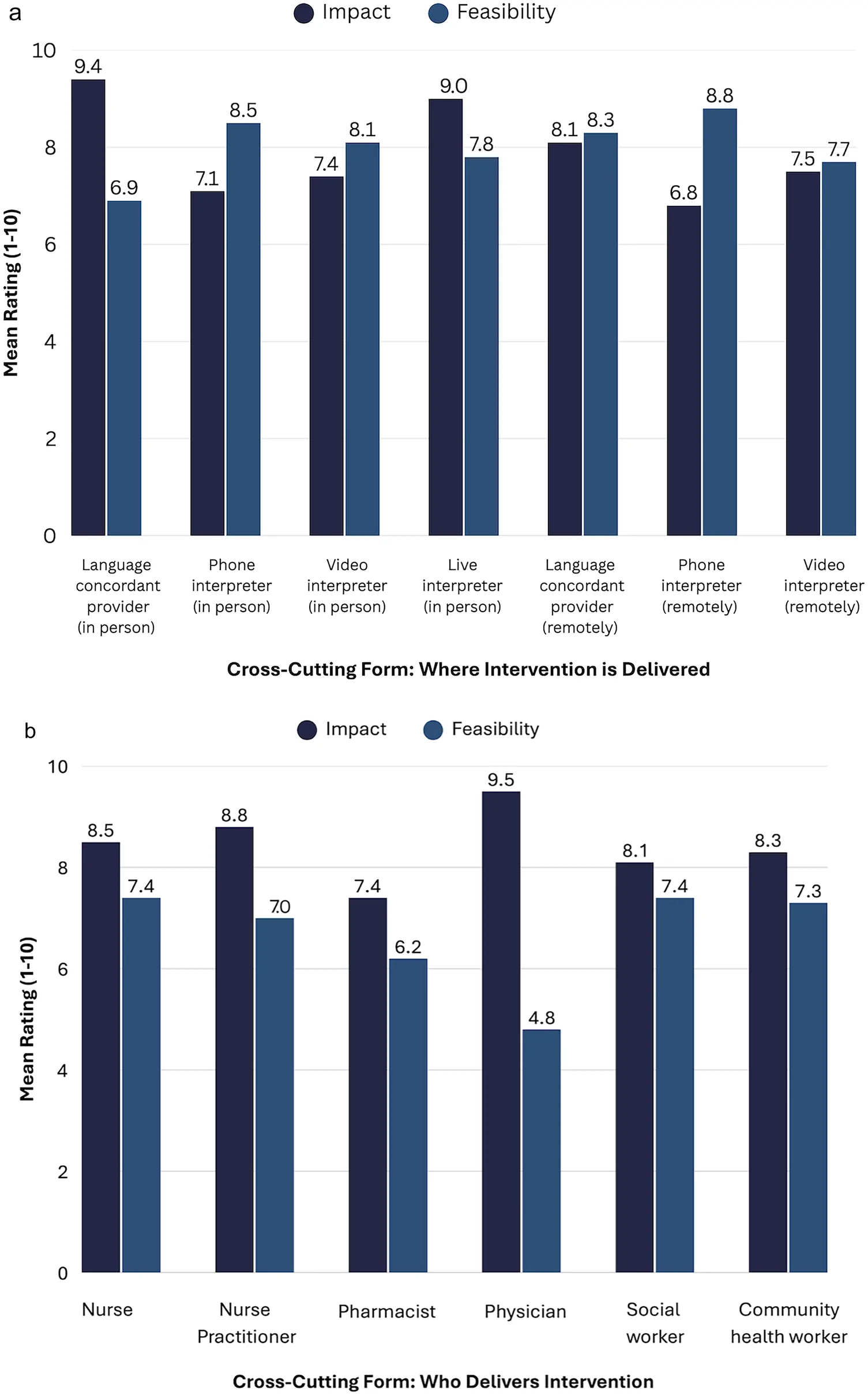

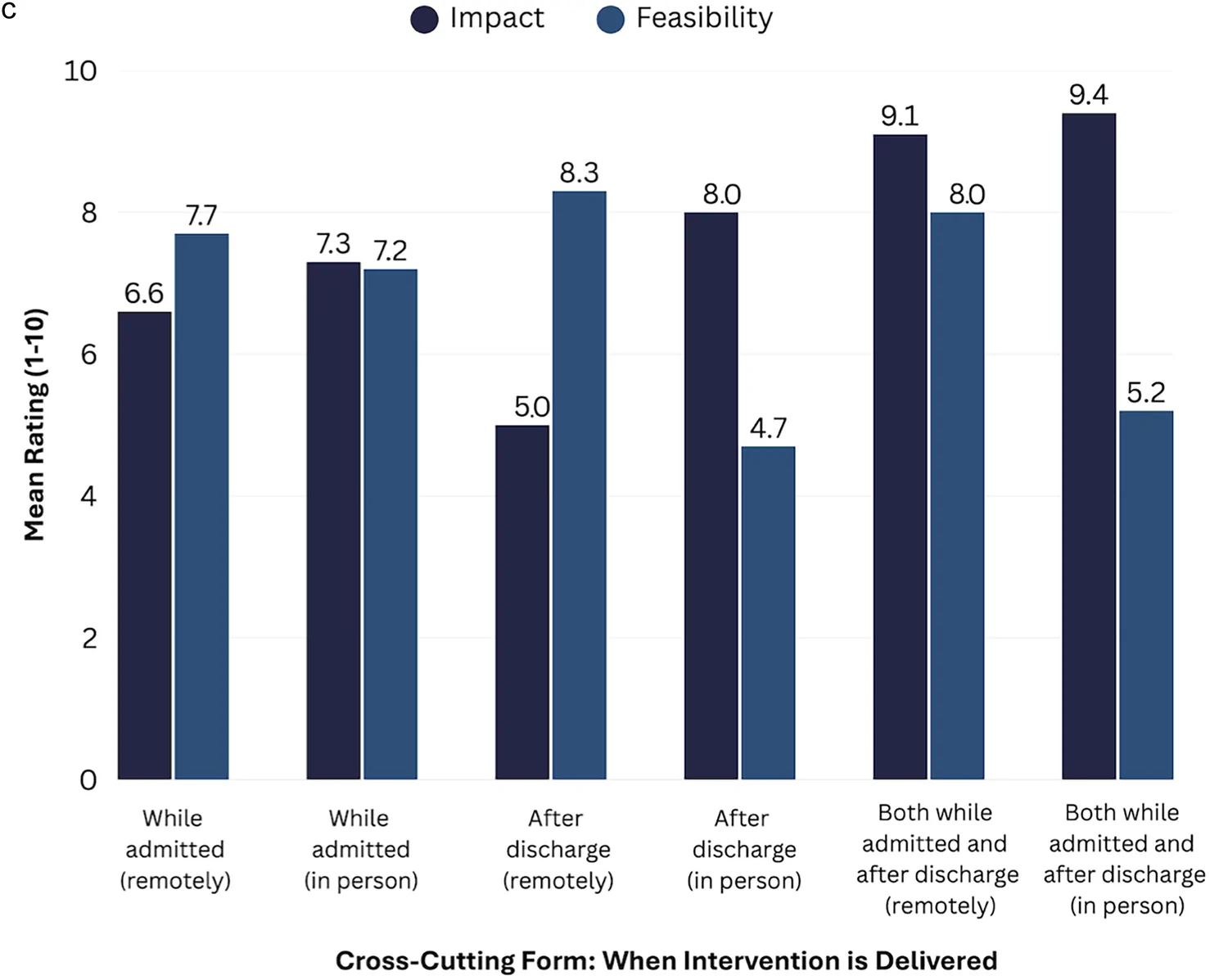
Feasibility and impact ratings of cross-cutting forms of hospital discharge interventions for patients with non-English language preference (NELP): who delivers the intervention **(a)**, when it is delivered **(b)**, and how the language barrier is overcome **(c)**.

For cross-cutting options on who should deliver the intervention (Figure 4a), physician delivery was rated highest for perceived impact (mean impact = 9.5) but lowest for feasibility (mean feasibility = 4.8). Nurses, social workers, and community health workers were also seen as highly impactful (mean impact range 8.1–8.5) while being considerably more feasible (mean feasibility range 7.3–7.4), suggesting these non-physician roles offer a better balance of effect and practicality.

For decisions about timing and location of delivery (Figure 4b), Adaptation Team members rated in–person approaches as having greater impact than remote modalities, and models that included contact both during the hospital stay and after discharge as more impactful than those limited to only one time period. The highest impact model was in-person contact during admission plus post–discharge follow–up (mean impact = 9.4), but it scored low on feasibility (mean feasibility = 4.3). By contrast, a remote-only post–discharge- model (in which interventionists connect with patients via phone or video rather than in person) received the highest feasibility rating (mean feasibility = 8.3) but a lower impact score (mean impact = 6.6), illustrating a clear tradeoff between expected impact and practicality.

For crosscutting options for achieving language concordance with diverse NELP participants (Figure 4c), team ratings favored in-person, language-concordant delivery as most impactful (mean impact = 9.4), closely followed by in-person delivery using a live interpreter (mean impact = 9.0). Phone delivery by a language concordant provider was rated as less impactful than in-person care (mean impact = 8.1) but more feasible (mean feasibility = 8.3). Remote delivery using an interpreter by phone or video scored lower on impact (mean impact = 6.8 and 7.5, respectively), indicating that language-concordant providers—especially in person—were seen as the most effective option, while telephone delivery by a concordant provider offered the best balance of impact and feasibility.

### Intervention forms

Table 3 links the specific forms suggested by the Adaptation Team for four of the Core Functions. For understanding and addressing patient priorities, examples include structured tools (such as a goalsetting worksheet or a strengths/needs assessment) and standardized documentation of patients' top three priorities and linguistic/cultural preferences. To promote disease understanding, suggested forms include language-concordant written instructions, links to educational videos in preferred language, peer educators, and post-discharge phone calls to reinforce instructions. To involve caregivers, recommendations focused on documenting the primary caregiver and scheduling discharge teaching when the caregiver can attend. To facilitate post-discharge communication, proposals included providing a direct post-discharge phone line, creating an online question forum, enrolling patients in the patient portal during admission, and offering hospital lobby computers for messaging access.

**Table 3:** Example of specific intervention forms suggested by adaptation team linked to core functions.

Core Function	Specific Forms
Understand and address patient priorities	- Create a document for patients to brainstorm health goals - Include in standardized documentation patient's top three priorities - Include in standardized documentation linguistic and cultural preferences - Develop a “strengths and needs” patient assessment tool
Promote understanding of disease	- Offer language concordant written discharge instructions - Provide links to educational videos in preferred language - Offer education from a peer - Telephone call post-discharge to reinforce written discharge instructions
Involve caregiver	- Document patient preferences around most important caregiver - Schedule discharge teaching at a time caregiver can be present
Facilitate post-discharge communication	- Provide a number to call after discharge that gives direct access to a provider - Create an online post-discharge question forum - Actively enroll patients in MyChart while admitted - Have computers in hospital lobby for patients to use for post-discharge messaging in case they do not have access to technology

## Discussion

The need for improved approaches to adapting complex multi-component interventions is well documented (86). In this manuscript we describe how the CFF model, integrated with the ADAPT methodology, guided our adaptation of hospital discharge interventions to better meet the needs of patients with NELP (11, 26). Framing adaptation around core functions (the causal processes that must be preserved to achieve intended effects) and adaptable forms (the context-sensitive ways to deliver those core functions) allowed us to translate shared functions across hospital discharge EBIs into delivery options tailored to those with NELP patients.

Although interest in the CFF model is growing, prospective, stakeholder engaged applications remain-limited (87–90). Unlike prior applications that described adaptations retrospectively, we developed a stepwise, prospective process for engaging stakeholders to identify and prioritize core functions and select context appropriate-forms (91–94). In this way, the CFF model complements ADAPT: ADAPT provides the steps for assessing fit and documenting changes, while the CFF model supplies explicit guidance on what to preserve (functions) vs. what can be varied (forms such as activities, materials, and delivery modes) to maintain mechanisms of effect across contexts (11, 95).

A novel feature of our methodology is applying the CFF model across a family of related EBIs rather than to a single program. We treated intervention adaptation and development as a continuum: infer core functions from existing hospital discharge interventions, prioritize those most likely to benefit the new target population, and then choose context-appropriate forms to deliver those functions. This function-to-form approach shows how shared mechanisms across different EBIs can be reused and tailored, enabling coordinated, scalable adaptations for interventions with common goals and components. After piloting the adapted intervention, we plan a hybrid effectiveness–implementation trial to confirm clinical benefit and continue evaluating implementation outcomes. Because our adaptation combines mechanisms and delivery features from multiple programs, such a trial is warranted in this case, but may not be in another application of the methodology where adaptations are less transformative.

To enhance operational usefulness, we subdivided forms into specific forms and crosscutting forms. This clarifies “form” by separating what is delivered from who delivers it, when/where, and how. Specific forms are concrete components (e.g., written discharge summaries, post-discharge calls); crosscutting forms are implementation features that span components (e.g., deliverer, timing, communication mode, language modality). This distinction was useful where a single core function could be achieved via multiple component-delivery combinations. Making specific and crosscutting forms explicit highlighted implementation decisions often omitted from intervention descriptions and prompted systematic consideration of linguistically relevant adaptations (e.g., in-person vs. remote delivery and how to achieve language concordance). These choices often determine-whether an intervention is accessible and effective for NELP patients.

A key drawback of the CFF model is that it does not systematically prompt adapters to anticipate unintended consequences of changing forms. Frameworks such as Model for Implementation Design and Impact (MADI) emphasize mapping downstream “ripple” effects and documenting potential mediators and moderators when delivery features are altered (21). For example, replacing in person, language-concordant delivery with interpreter-mediated-phone encounters may still convey key discharge information (core function), but can also reduce patient engagement, erode trust, and limit nonverbal communication (96). Those downstream effects may blunt the intervention's mechanism (e.g., patient activation or comprehension) and ultimately reduce its effectiveness counter to the intention of adaptation efforts. When correctly applied the CFF model should help adapters know through measurement of core functions if it was achieved or not; without complementary attention to causal pathways it may not explain why a form change produced that failure.

A central challenge for the CFF model and other mechanism focused adaptation frameworks is reliably identifying the mechanisms themselves. Guidance calls for explicit attention to theory or mechanisms of action and for preserving “core components,” yet many trials do not define or justify those elements (1). In our case study we relied on a preliminary list of core functions derived from a structured literature review and additional input from the Adaptation Team, similar to others (97). An alternative and more rigorous approach would be to use a formal consensus process (e.g., Delphi) to engage a broad group of experts in hospital discharge intervention including social workers, physicians, and nurses to define functions (93, 98). As the field advances, aligning terms such as “core components,” “mechanisms,” and “core functions” across intervention development, implementation science, and adaptation will improve consistency and guide methodological choices (88, 99, 100).

An additional limitation concerns the level of abstraction at which core functions should be specified, as prior applications of the CFF model have varied in granularity. For example, in applying it to the patient-centered medical home, Perez Jolles et al. nested multiple core functions within an overarching principle (e.g., “accessible care”) (26). In our application, some elements, such as “involve caregivers,” could reasonably be conceptualized as higher-level principles rather than core functions, with more specific operationalizations articulated beneath them. Alternative structuring choices may have resulted in a different set or hierarchy of core functions. Future work should further clarify guidance on how to define and operationalize core functions to enhance consistency and comparability across studies.

Two additional limitations should be noted. First, we did not employ a formal Delphi process with predefined consensus thresholds. This was a deliberate choice: the Delphi technique is expressly designed to prevent direct interaction among panelists, whereas dialogue among patients, caregivers, and clinicians was central to our codesign approach. Ranking and rating exercises were therefore used to surface divergence and focus discussion rather than serve as decision rules. We acknowledge this limits comparability with consensus derived through established Delphi methods (101). Future efforts could consider the nominal group technique or equity-focused modifications to the Delphi process to promote equitable participation (102–104). Second, the Adaptation Team skewed female and predominantly comprised health care workers—including several members recruited to represent patient and caregiver perspectives—narrowing the range of lived experience captured and potentially shaping which core functions were prioritized and how they were interpreted. This was partially mitigated by the team's composition: 54% spoke a language other than English at home and 85% had caregiving experience. Nonetheless, professionally embedded perspectives may not fully reflect those of patients with NELP navigating discharge without clinical knowledge or institutional familiarity.

The approach we present here has important implications for health equity. Patients with NELP face many barriers to EBIs and have disproportionately high risk of communication failures at discharge and adverse outcomes such as unplanned readmissions (32, 48), yet have largely been excluded from prior discharge trials (40, 41). Creating entirely new interventions for each language group would be costly and underuse existing evidence (105). Health disparities scholars have warned of “sideways progress,” where repeated, intensive adaptations for each demographic subgroup consume resources without producing broadly scalable solutions (106). Our approach seeks efficiency and real-world-relevance by (1) building on proven discharge EBIs and making targeted adaptations needed for NELP populations and (2) adapting for the broader NELP group rather than designing separate programs for each language or cultural subgroup, aligning adaptations with diverse clinical settings to improve feasibility and scalability (10).

## Conclusions

Integrating the CFF model with the ADAPT process offers a practical and theory-grounded approach for equity-focused adaptation of complex, multicomponent interventions and effectively facilitated adaptation by drawing from the numerous hospital discharge interventions tested to date. By specifying measurable core functions and distinguishing between specific and cross-cutting forms, this combined approach helps implementers preserve intervention mechanisms while tailoring delivery to the linguistic and cultural needs of populations with NELP. Applying these methods to a family of related discharge interventions, rather than to a single program, illustrates how shared mechanisms can be translated into context-sensitive delivery options that are both actionable for health systems and amenable to rigorous evaluation. For practice, the core functions–forms distinction gives frontline teams a transferable way to adapt the surface features of a discharge intervention to the patients in front of them while safeguarding the elements that drive outcomes, rather than relying on *ad hoc* or improvised adjustments. For health equity, this approach makes rigorous adaptation feasible at scale for populations historically excluded from discharge trials, helping ensure that the benefits of proven EBIs reach NELP patients rather than stopping at the boundary of the languages and settings in which they were first tested. Future work should refine guidance on defining core functions and test this approach across diverse settings and populations.

## Data Availability

The raw data supporting the conclusions of this article will be made available by the authors, without undue reservation.

## References

[1] Movsisyan A Arnold L Copeland L Evans R Littlecott H Moore G. Adapting evidence-informed population health interventions for new contexts: a scoping review of current practice. Health Res Policy Syst. (2021) 19(1):13. 10.1186/s12961-020-00668-9 33546707 PMC7863549

[2] Kumpfer K Magalhães C Xie J. Cultural adaptation and implementation of family evidence-based interventions with diverse populations. Prev Sci. (2017) 18(6):649–59. 10.1007/s11121-016-0719-3 27757773

[3] Jones JM. Culturally responsive adaptations to evidence-based interventions for black adolescents. Sch Psychol. (2025) 40(2):274–85. 10.1037/spq0000688 40193509

[4] Castro FG Yasui M. Advances in EBI development for diverse populations: towards a science of intervention adaptation. Prev Sci. (2017) 18(6):623–9. 10.1007/s11121-017-0809-x 28620723 PMC5667940

[5] Arundell LL Barnett P Buckman JEJ Saunders R Pilling S. The effectiveness of adapted psychological interventions for people from ethnic minority groups: a systematic review and conceptual typology. Clin Psychol Rev. (2021) 88:102063. 10.1016/j.cpr.2021.102063 34265501 PMC8591374

[6] Healey P Stager ML Woodmass K Dettlaff AJ Vergara A Janke R. Cultural adaptations to augment health and mental health services: a systematic review. BMC Health Serv Res. (2017) 17(1):1–26. 10.1186/s12913-016-1953-x 28056967 PMC5217593

[7] Griner D Smith TB. Culturally adapted mental health intervention: a meta-analytic review. Special issue: culture, race, and ethnicity in psychotherapy. Psychotherapy. (2006) 43(4):531–48. 10.1037/0033-3204.43.4.531 22122142

[8] Chowdhary N Jotheeswaran AT Nadkarni A Hollon SD King M Jordans MJD. The methods and outcomes of cultural adaptations of psychological treatments for depressive disorders: a systematic review. Psychol Med. (2014) 44(6):1131–46. 10.1017/S0033291713001785 23866176 PMC3943384

[9] Barrera M Berkel C Castro FG. Directions for the advancement of culturally adapted preventive interventions: local adaptations, engagement, and sustainability. Prev Sci. (2017) 18(6):640–8. 10.1007/s11121-016-0705-9 27591993 PMC7678089

[10] Austad K Cordova-Ramos EG Fernandez A Drainoni ML. Lost in translation: advancing intervention adaptation for populations with non-dominant language preference in high diversity settings. Implement Sci Commun. (2025) 6(1):66. 10.1186/s43058-025-00753-6 40437645 PMC12117692

[11] Moore G Campbell M Copeland L Craig P Movsisyan A Hoddinott P. Adapting interventions to new contexts-the ADAPT guidance. BMJ. (2021) 374:n1679. 10.1136/bmj.n1679 34344699 PMC8329746

[12] Movsisyan A Arnold L Evans R Hallingberg B Moore G O’Cathain A. Adapting evidence-informed complex population health interventions for new contexts: a systematic review of guidance. Implement Sci. (2019) 14(1):105. 10.1186/s13012-019-0956-5 31847920 PMC6918624

[13] Davis T DiClemente RJ Prietula M. Using ADAPT-ITT to modify a telephone-based HIV prevention intervention for SMS delivery: formative study. JMIR Form Res. (2020) 4:e22485. 10.2196/22485 32831178 PMC7576465

[14] Damschroder LJ Aron DC Keith RE Kirsh SR Alexander JA Lowery JC. Fostering implementation of health services research findings into practice: a consolidated framework for advancing implementation science. Implement Sci. (2009) 4(1):50. 10.1186/1748-5908-4-50 19664226 PMC2736161

[15] Chambers DA. Advancing adaptation of evidence-based interventions through implementation science: progress and opportunities. Front Health Serv. (2023) 3:1204138. 10.3389/frhs.2023.1204138 37342795 PMC10277471

[16] Chambers DA Norton WE. The adaptome: advancing the science of intervention adaptation. Am J Prev Med. (2016) 51(4):S124–31. 10.1016/j.amepre.2016.05.011 27371105 PMC5030159

[17] Castro FG Barrera M Martinez CR. The cultural adaptation of prevention interventions: resolving tensions between fidelity and fit. Prev Sci. (2004) 5(1):41–5. 10.1023/b:prev.0000013980.12412.cd 15058911

[18] CFIR Research Team, Team CR, CFIR Research Team. Consolidated framework for implementation research (2014). Available online at: http://www.cfirguide.org/ (Accessed February 20, 2026).

[19] Minary L Alla F Cambon L Kivits J Potvin L. Addressing complexity in population health intervention research: the context/intervention interface. J Epidemiol Community Health. (2018) 72(4):319–23. 10.1136/jech-2017-209921 29321174 PMC5868525

[20] Nilsen P. Making sense of implementation theories, models and frameworks. Implement Sci. (2015) 10(1):1–13. 10.1186/s13012-015-0242-0 25895742 PMC4406164

[21] Kirk MA Moore JE Wiltsey Stirman S Birken SA. Towards a comprehensive model for understanding adaptations’ impact: the model for adaptation design and impact (MADI). Implement Sci. (2020) 15(1):56. 10.1186/s13012-020-01021-y 32690104 PMC7370455

[22] Wiedenman EM Campbell-Scherer D Iqbal A Ofosu NN Hyde AM McKay RC. Is it time for a paradigm shift to complexity-informed implementation fidelity? Npj Complex. (2025) 2(1):34. 10.1038/s44260-025-00057-9

[23] Shepherd-Banigan M Goldstein KM Boucher NA McConnell ES McDermott CL Ma JE. Characterizing intervention components and complexity of nonpharmacologic healthcare interventions to manage distress behaviors in older adults. J Appl Gerontol. (2026) 45(1):166–76. 10.1177/07334648251330673 40294900 PMC12353672

[24] Esmail LC Barasky R Mittman BS Hickam DH. Improving comparative effectiveness research of complex health interventions: standards from the Patient-Centered Outcomes Research Institute (PCORI). J Gen Intern Med. (2020) 35(S2):875–81. 10.1007/s11606-020-06093-6 33107006 PMC7652976

[25] Hawe P Shiell A Riley T. Complex interventions: how “out of control” can a randomised controlled trial be? Br Med J. (2004) 328(7455):1561–3. 10.1136/bmj.328.7455.1561 15217878 PMC437159

[26] Perez Jolles M Lengnick-Hall R Mittman BS. Core functions and forms of complex health interventions: a patient-centered medical home illustration. J Gen Intern Med. (2019) 34(6):1032–8. 10.1007/s11606-018-4818-7 30623387 PMC6544719

[27] Hill J Cuthel AM Lin P Grudzen CR. Primary palliative care for emergency medicine (PRIM-ER): applying form and function to a theory-based complex intervention. Contemp Clin Trials Commun. (2020) 18:100570. 10.1016/j.conctc.2020.100570 32426550 PMC7225617

[28] Howard AF Lynch K Thorne S Currie LM Arora RC McDermid RC. From hospital to home: a heightened window of vulnerability post-critical illness. PLoS One. (2025) 20(10):e0334092. 10.1371/journal.pone.0334092 41060985 PMC12507260

[29] Daliri S Bekker CL Buurman BM Scholte Op Reimer WJM Van Den Bemt BJF Karapinar-Çarkit F. Barriers and facilitators with medication use during the transition from hospital to home: a qualitative study among patients. BMC Health Serv Res. (2019) 19(1):204. 10.1186/s12913-019-4028-y 30925880 PMC6441233

[30] van Grootel JWM Collet RJ Van Dongen JM Van Der Leeden M Geleijn E Ostelo R. Experiences with hospital-to-home transitions: perspectives from patients, family members and healthcare professionals. A systematic review and meta-synthesis of qualitative studies. Disabil Rehabil. (2025) 47(7):1644–58. 10.1080/09638288.2024.2384624 39101687 PMC11974919

[31] Jehloh L Songwathana P Kitrungrote L Bourbonnais A. Perspectives of family caregivers and nurses on hospital discharge transitional care for muslim older adults living with COPD: a qualitative study. BMC Nurs. (2024) 23(1):273. 10.1186/s12912-024-01943-8 38659051 PMC11044287

[32] Squires A Ma C Miner S Feldman P Jacobs EA Jones SA. Assessing the influence of patient language preference on 30 day hospital readmission risk from home health care: a retrospective analysis. Int J Nurs Stud. (2022) 125:104093. 10.1016/j.ijnurstu.2021.104093 34710627 PMC9282680

[33] Jack BW Chetty VK Anthony D Greenwald JL Sanchez GM Johnson AE. A reengineered hospital discharge program to decrease rehospitalization: a randomized trial. Ann Intern Med. (2009) 150:178–87. 10.7326/0003-4819-150-3-200902030-00007 19189907 PMC2738592

[34] Berkowitz RE Fang Z Helfand BKI Jones RN Schreiber R Paasche-Orlow MK. Project ReEngineered Discharge (RED) lowers hospital readmissions of patients discharged from a skilled nursing facility. J Am Med Dir Assoc. (2013) 14(10):736–40. 10.1016/j.jamda.2013.03.004 23608528

[35] Puhr MI Thompson HJ. The use of transitional care models in patients with stroke. J Neurosci Nurs. (2015) 47(4):223–34. 10.1097/JNN.0000000000000143 25906245

[36] Hansen LO Greenwald JL Budnitz T Howell E Halasyamani L Maynard G. Project BOOST: effectiveness of a multihospital effort to reduce rehospitalization. J Hosp Med. (2013) 8(8):421–7. 10.1002/jhm.2054 23873709

[37] Coleman EA Parry C Chalmers S Min S-J. The care transitions intervention: results of a randomized controlled trial. Arch Intern Med. (2006) 166(17):1822–8. 10.1001/archinte.166.17.1822 17000937

[38] Becker C Zumbrunn S Beck K Vincent A Loretz N Müller J. Interventions to improve communication at hospital discharge and rates of readmission: a systematic review and meta-analysis. JAMA Netw Open. (2021) 4(8):e2119346. 10.1001/jamanetworkopen.2021.19346 34448868 PMC8397933

[39] Tyler N Hodkinson A Planner C Angelakis I Keyworth C Hall A. Transitional care interventions from hospital to community to reduce health care use and improve patient outcomes: a systematic review and network meta-analysis. JAMA Netw Open. (2023) 6(11):e2344825. 10.1001/jamanetworkopen.2023.44825 38032642 PMC10690480

[40] Oyesanya TO Loflin C Byom L Harris G Daly K Rink L. Transitions of care interventions to improve quality of life among patients hospitalized with acute conditions: a systematic literature review. Health Qual Life Outcomes. (2021) 19(1):36. 10.1186/s12955-021-01672-5 33514371 PMC7845026

[41] Sezgin D O’Caoimh R Liew A O’Donovan MR Illario M Salem MA. The effectiveness of intermediate care including transitional care interventions on function, healthcare utilisation and costs: a scoping review. Eur Geriatr Med. (2020) 11(6):961–74. 10.1007/s41999-020-00365-4 32754841 PMC7402396

[42] Ortman JM Shin HB. (2011, August 20–23). Language projections: 2010 to 2020 [Conference paper]. Annual Meeting of the American Sociological Association. Las Vegas, NV: U.S. Census Bureau.

[43] Austad K Jack BW. Linguistic and cultural competence at hospital discharge. J Healthc Manag Stand. (2023) 3(1):1–16. 10.4018/JHMS.330644

[44] Choe AY Unaka NI Schondelmeyer AC Bignall WJR Vilvens HL Thomson JE. Inpatient communication barriers and drivers when caring for limited English proficiency children. J Hosp Med. (2019) 14(10):607–13. 10.12788/jhm.3240 31339836 PMC6817305

[45] Davis SH Rosenberg J Nguyen J Jimenez M Lion KC Jenicek G. Translating discharge instructions for limited English–proficient families: strategies and barriers. Hosp Pediatr. (2019) 9(10):779–87. 10.1542/hpeds.2019-0055 31562199 PMC6876295

[46] Choe AY Schondelmeyer AC Thomson J Schwieter A McCann E Kelley J. Improving discharge instructions for hospitalized children with limited English proficiency. Hosp Pediatr. (2021) 11(11):1213–22. 10.1542/hpeds.2021-005981 34654727

[47] Pandey M Maina RG Amoyaw J Li Y Kamrul R Michaels CR. Impacts of English language proficiency on healthcare access, use, and outcomes among immigrants: a qualitative study. BMC Health Serv Res. (2021) 21(1):741. 10.1186/s12913-021-06750-4 34311712 PMC8314461

[48] Malevanchik L Wheeler M Gagliardi K Karliner L Shah SJ. Disparities after discharge: the association of limited English proficiency and postdischarge patient-reported issues. Jt Comm J Qual Patient Saf. (2021) 47(12):775–82. 10.1016/j.jcjq.2021.08.013 34627715 PMC9246478

[49] Karliner LS Auerbach A Nápoles A Schillinger D Nickleach D Pérez-Stable EJ. Language barriers and understanding of hospital discharge instructions. Med Care. (2012) 50(4):283–9. 10.1097/MLR.0b013e318249c949 22411441 PMC3311126

[50] Balaban RB Weissman JS Samuel PA Woolhandler S. Redefining and redesigning hospital discharge to enhance patient care: a randomized controlled study. J Gen Intern Med. (2008) 23(8):1228–33. 10.1007/s11606-008-0618-9 18452048 PMC2517968

[51] Bell SP Schnipper JL Goggins K Bian A Shintani A Roumie CL. Effect of pharmacist counseling intervention on health care utilization following hospital discharge: a randomized control trial. J Gen Intern Med. (2016) 31(5):470–7. 10.1007/s11606-016-3596-3 26883526 PMC4835388

[52] Biese KJ Busby-Whitehead J Cai J Stearns SC Roberts E Mihas P. Telephone follow-up for older adults discharged to home from the emergency department: a pragmatic randomized controlled trial. J Am Geriatr Soc. (2018) 66(3):452–8. 10.1111/jgs.15142 29272029

[53] Bloodworth LS Malinowski SS Lirette ST Ross LA. Pharmacist linkage in care transitions: from academic medical center to community. J Am Pharm Assoc. (2019) 59(6):896–904. 10.1016/j.japh.2019.08.011 31590926

[54] Bloom SL Stollings JL Kirkpatrick O Wang L Byrne DW Sevin CM. Randomized clinical trial of an ICU recovery pilot program for survivors of critical illness. Crit Care Med. (2019) 47(10):1337–45. 10.1097/CCM.0000000000003909 31385881

[55] Blum K Gottlieb SS. The effect of a randomized trial of home telemonitoring on medical costs, 30-day readmissions, mortality, and health-related quality of life in a cohort of community-dwelling heart failure patients. J Card Fail. (2014) 20(7):513–21. 10.1016/j.cardfail.2014.04.016 24769270

[56] Dalal AK Schaffer A Gershanik EF Papanna R Eibensteiner K Nolido NV. The impact of automated notification on follow-up of actionable tests pending at discharge: a cluster-randomized controlled trial. J Gen Intern Med. (2018) 33(7):1043–51. 10.1007/s11606-018-4393-y 29532297 PMC6025668

[57] Dawson NL Hull BP Vijapura P Dumitrascu AG Ball CT Thiemann KM. Home telemonitoring to reduce readmission of high-risk patients: a modified intention-to-treat randomized clinical trial. J Gen Intern Med. (2021) 36(11):3395–401. 10.1007/s11606-020-06589-1 33506388 PMC8606403

[58] DeVore AD Granger BB Fonarow GC Al-Khalidi HR Albert NM Lewis EF. Effect of a hospital and postdischarge quality improvement intervention on clinical outcomes and quality of care for patients with heart failure with reduced ejection fraction: the CONNECT-HF randomized clinical trial. JAMA. (2021) 326(4):314–23. 10.1001/jama.2021.8844 34313687 PMC8317015

[59] Englander H Michaels L Chan B Kansagara D. The care transitions innovation (C-TRAIN) for socioeconomically disadvantaged adults: results of a cluster randomized controlled trial. J Gen Intern Med. (2014) 29(11):1460–7. 10.1007/s11606-014-2903-0 24913003 PMC4238212

[60] Evans RL Hendricks RD. Evaluating hospital discharge planning: a randomized clinical trial. Med Care. (1993) 31(4):358–70. 10.1097/00005650-199304000-00007 8464252

[61] Farris KB Carter BL Xu Y Dawson JD Shelsky C Weetman DB. Effect of a care transition intervention by pharmacists: an RCT. BMC Health Serv Res. (2014) 14:406. 10.1186/1472-6963-14-406 25234932 PMC4262237

[62] Gardner RL Pelland K Youssef R Morphis B Calandra K Hollands L. Reducing hospital readmissions through a skilled nursing facility discharge intervention: a pragmatic trial. J Am Med Dir Assoc. (2020) 21(4):508–12. 10.1016/j.jamda.2019.10.001 31812334

[63] Goldman LE Sarkar U Kessell E Guzman D Schneidermann M Pierluissi E. Support from hospital to home for elders: a randomized trial. Ann Intern Med. (2014) 161(7):472–81. 10.7326/M14-0094 25285540

[64] Gurwitz JH Field TS Ogarek J Tjia J Cutrona SL Harrold LR. An electronic health record-based intervention to increase follow-up office visits and decrease rehospitalization in older adults. J Am Geriatr Soc. (2014) 62(5):865–71. 10.1111/jgs.12798 24779524 PMC4351873

[65] Kennedy L Neidlinger S Scroggins K. Effective comprehensive discharge planning for hospitalized elderly. Gerontologist. (1987) 27(5):577–80. 10.1093/geront/27.5.577 3678895

[66] Kripalani S Roumie CL Dalal AK Cawthon C Businger A Eden SK. Effect of a pharmacist intervention on clinically important medication errors after hospital discharge: a randomized trial. Ann Intern Med. (2012) 157(1):1–10. 10.7326/0003-4819-157-1-201207030-00003 22751755 PMC3575734

[67] Laramee AS Levinsky SK Sargent J Ross R Callas P. Case management in a heterogeneous congestive heart failure population: a randomized controlled trial. Arch Intern Med. (2003) 163(7):809–17. 10.1001/archinte.163.7.809 12695272

[68] Levine DM Ouchi K Blanchfield B Diamond K Licurse A Pu CT. Hospital-Level care at home for acutely ill adults: a pilot randomized controlled trial. J Gen Intern Med. (2018) 33(5):729–36. 10.1007/s11606-018-4307-z 29411238 PMC5910347

[69] Magny-Normilus C Nolido NV Borges JC Brady M Labonville S Williams D. Effects of an intensive discharge intervention on medication adherence, glycemic control, and readmission rates in patients with type 2 diabetes. J Patient Saf. (2021) 17(2):73–80. 10.1097/PTS.0000000000000601 31009408 PMC7647006

[70] McWilliams A Roberge J Anderson WE Moore CG Rossman W Murphy S. Aiming to improve readmissions through integrated hospital transitions (AIRTIGHT): a pragmatic randomized controlled trial. J Gen Intern Med. (2019) 34(1):58–64. 10.1007/s11606-018-4617-1 30109585 PMC6318199

[71] Naylor M Brooten D Jones R Lavizzo-Mourey R Mezey M Pauly M. Comprehensive discharge planning for the hospitalized elderly: a randomized clinical trial. Ann Intern Med. (1994) 120(12):999–1006. 10.7326/0003-4819-120-12-199406150-00005 8185149

[72] Noel K Messina C Hou W Schoenfeld E Kelly G. Tele-transitions of care (TTOC): a 12-month, randomized controlled trial evaluating the use of telehealth to achieve triple aim objectives. BMC Fam Pr. (2020) 21(1):27. 10.1186/s12875-020-1094-5 PMC7007639 32033535

[73] Ong MK Romano PS Edgington S Aronow HU Auerbach AD Black JT. Effectiveness of remote patient monitoring after discharge of hospitalized patients with heart failure: the better effectiveness after transition-heart failure (BEAT-HF) randomized clinical trial. JAMA Intern Med. (2016) 176(3):310–8. 10.1001/jamainternmed.2015.7712 26857383 PMC4827701

[74] Phatak A Prusi R Ward B Hansen LO Williams MV Vetter E. Impact of pharmacist involvement in the transitional care of high-risk patients through medication reconciliation, medication education, and postdischarge call-backs (IPITCH Study). J Hosp Med. (2016) 11(1):39–44. 10.1002/jhm.2493 26434752

[75] Piette JD Striplin D Aikens JE Lee A Marinec N Mansabdar M. Impacts of post-hospitalization accessible health technology and caregiver support on 90-day acute care use and self-care assistance: a randomized clinical trial. Am J Med Qual. (2021) 36(3):145–55. 10.1177/1062860620943673 32723072

[76] Rich MW Vinson JM Sperry JC Shah AS Spinner LR Chung MK. Prevention of readmission in elderly patients with congestive heart failure: results of a prospective, randomized pilot study. J Gen Intern Med. (1993) 8(11):585–90. 10.1007/BF02599709 8289096

[77] Rich MW Beckham V Wittenberg C Leven CL Freedland KE Carney RM. A multidisciplinary intervention to prevent the readmission of elderly patients with congestive heart failure. N Engl J Med. (1995) 333(18):1190–5. 10.1056/NEJM199511023331806 7565975

[78] Ritchie CS Houston TK Richman JS Sobko HJ Berner ES Taylor BB. The e-coach technology-assisted care transition system: a pragmatic randomized trial. Transl Behav Med. (2016) 6(3):428–37. 10.1007/s13142-016-0422-8 27339715 PMC4987612

[79] Sales VL Ashraf MS Lella LK Huang J Bhumireddy G Lefkowitz L. Utilization of trained volunteers decreases 30-day readmissions for heart failure. J Card Fail. (2013) 19(12):842–50. 10.1016/j.cardfail.2013.10.008 24331204

[80] Strano A Briggs A Powell N Brockman J Taylor J Butler A. Home healthcare visits following hospital discharge: does the timing of visits affect 30-day hospital readmission rates for heart failure patients? Home Heal Now. (2019) 37(3):152–7. 10.1097/NHH.0000000000000740 31058733

[81] Vinluan CM Wittman D Morisky D. Effect of pharmacist discharge counselling on medication adherence in elderly heart failure patients: a pilot study: pharmacist discharge counselling in the elderly. J Pharm Health Serv Res. (2015) 6(2):103–10. 10.1111/jphs.12093

[82] Weiss ME Yakusheva O Bobay KL Costa L Hughes RG Nuccio S. Effect of implementing discharge readiness assessment in adult medical-surgical units on 30-day return to hospital: the READI randomized clinical trial. JAMA Netw Open. (2019) 2(1):e187387. 10.1001/jamanetworkopen.2018.7387 30681712 PMC6484543

[83] Mentimeter. (2025). Available online at: https://www.mentimeter.com/ (Accessed February 20, 2026).

[84] Dalkey N Helmer O. An experimental application of the DELPHI method to the use of experts. Manag Sci. (1963) 9(3):458–67. 10.1287/mnsc.9.3.458

[85] Wiltsey Stirman S Baumann AA Miller CJ. The FRAME: an expanded framework for reporting adaptations and modifications to evidence-based interventions. Implement Sci. (2019) 14(1):1–10. 10.1186/s13012-019-0898-y 31171014 PMC6554895

[86] Uddin J Joshi VL Wells V Faruque M Mashreky SR Movsisyan A. Adaptation of complex interventions for people with long-term conditions: a scoping review. Transl Behav Med. (2024) 14(9):514–26. 10.1093/tbm/ibae031 38895875 PMC11370634

[87] Terrana A Viglione C Rhee K Rabin B Godino J Aarons GA. The core functions and forms paradigm throughout EPIS: designing and implementing an evidence-based practice with function fidelity. Front Health Serv. (2024) 3:1281690. 10.3389/frhs.2023.1281690 38292916 PMC10826509

[88] Fox K Passey D Kang E Zeigen L Kenzie E Austin JD. Application of core functions and forms in complex health intervention research: a scoping review protocol. BMJ Open. (2025) 15(1):e091088. 10.1136/bmjopen-2024-091088 39779266 PMC11749525

[89] Austin JD Morrill KE Kenzie E Fox K Hernandez-Ramirez RU Leeman J. Understanding Functions and Forms of Complex/Multilevel Interventions. Consortium for Cancer Implementation Science, Implementation of Complex/Multilevel Interventions Task Group; March 2023.

[90] Harrison MS. Functions and forms framework: a methodology for mechanistic deconstruction and adaptation? J Gen Intern Med. (2021) 36(9):2809–11. 10.1007/s11606-020-06533-3 33469751 PMC8390705

[91] Kirk MA Haines ER Rokoske FS Powell BJ Weinberger M Hanson LC. A case study of a theory-based method for identifying and reporting core functions and forms of evidence-based interventions. Transl Behav Med. (2021) 11(1):21–33. 10.1093/tbm/ibz178 31793635 PMC7877297

[92] Ferrari RM Randolph CM O’Leary MC Lich KH Moore AA Leeman J. What makes patient navigation work? Identifying functions and forms and conducting causal loop diagramming to specify components of a successful colorectal cancer patient navigation program. Implement Sci Commun. (2026) 7(1):32. 10.1186/s43058-026-00858-6 41549310 PMC12903651

[93] Juckett LA Bernard KP Clark MA Gadbois EA Wright B Thomas KS. Core functions and forms in home-delivered meal programs: a stakeholder-driven approach to identifying essential practices. Implement Sci Commun. (2025) 6(1):43. 10.1186/s43058-025-00728-7 40229891 PMC11998402

[94] Cervantes L Rizzolo K Ressalam J Maw A Golestaneh L Tuot DS. Adaptation of navigate-kidney: a community health worker intervention for people with kidney disease. Kidney Int Rep. (2026) 11:62–75. 10.1016/j.ekir.2025.10.011 41541772 PMC12799562

[95] Moore G Campbell M Copeland L Craig P Movsisyan A Hoddinott P. (2020). Adaptation of interventions for implementation and/or re-evaluation in new contexts: The ADAPT Study guidance (Version 1.0). DECIPHer Centre. Available online at: https://decipher.uk.net/portfolio/the-adapt-study (Accessed February 20, 2026).

[96] Heath M Hvass AMF Wejse CM. Interpreter services and effect on healthcare—a systematic review of the impact of different types of interpreters on patient outcome. J Migr Health. (2023) 7:100162. 10.1016/j.jmh.2023.100162 36816444 PMC9932446

[97] Patel J Shrestha S Marr R Caseley P Mack M Singh V. Identifying core functions and forms of “discharge by noon” interventions. J Gen Intern Med. (2025) 40(8):1910–6. 10.1007/s11606-024-09257-w 39838254 PMC12119409

[98] Thangaratinam S Redman CW. The Delphi technique. Obstet Gynaecol. (2005) 7(2):120–5. 10.1576/toag.7.2.120.27071

[99] Blase K Fixsen D. Core Intervention Components: Identifying and Operationalizing What Makes Program Work [Internet]. Office of the Assistant Secretary for Planning and Evaluation–U.S. Department of Health and Human Services. Washington DC: ASPE Research Brief (2013). Available online at: https://aspe.hhs.gov/reports/core-intervention-components-identifying-operationalizing-what-makes-programs-work-0 (Accessed February 20, 2026).

[100] Fixsen DL Blase KA Naoom SF Wallace F. Core implementation components. Res Soc Work Pract. (2009) 19(5):531–40. 10.1177/1049731509335549

[101] Li J Brock J Jack B Mittman B Naylor M Sorra J. Project ACHIEVE—using implementation research to guide the evaluation of transitional care effectiveness organization, structure and delivery of healthcare. BMC Health Serv Res. (2016) 16(1):1–9. 10.1186/s12913-016-1312-y 26896024 PMC4759940

[102] Cary MA Plamondon K Banner-Lukaris D Oelke N Sibley KM Baxter K. Building consensus in research partnerships: a scoping review of consensus methods. Evid Policy. (2023) 19(3):485–511. 10.1332/174426421X16645354235140

[103] Rankin NM McGregor D Butow PN White K Phillips JL Young JM. Adapting the nominal group technique for priority setting of evidence-practice gaps in implementation science. BMC Med Res Methodol. (2016) 16(1):110. 10.1186/s12874-016-0210-7 27566679 PMC5002198

[104] Niederberger M Sonnberger M. The participation of lifeworld experts in Delphi processes: a reflection on method and practice. MethodsX. (2025) 14:103274. 10.1016/j.mex.2025.103274 40230552 PMC11995758

[105] Alvidrez J Nápoles AM Bernal G Lloyd J Cargill V Godette D. Building the evidence base to inform planned intervention adaptations by practitioners serving health disparity populations. Am J Public Health. (2019) 109(S1):S94–101. 10.2105/AJPH.2018.304915 30699023 PMC6356120

[106] Alvidrez J Stinson N. Sideways progress in intervention research is not sufficient to eliminate health disparities. Am J Public Health. (2019) 109(S1):S102–4. 10.2105/AJPH.2019.304953 30699028 PMC6356126

